# Are food patterns associated with prostate cancer in Jamaican men: a preliminary report

**DOI:** 10.1186/1750-9378-4-S1-S5

**Published:** 2009-02-10

**Authors:** Maria Jackson, Susan Walker, Candace Simpson, Norma McFarlane-Anderson, Franklyn Bennett

**Affiliations:** 1 Dept. of Community Health and Psychiatry, University of the West Indies, Mona, Kingston, Jamaica; 2 Tropical Medicine Research Institute, University of the West Indies, Mona, Kingston, Jamaica; 3 Dept. of Basic Medical Sciences, University of the West Indies, Mona, Kingston, Jamaica; 4 Dept. of Pathology, University of the West Indies, Mona, Kingston, Jamaica

## Abstract

**Background:**

Morbidity and mortality data highlight prostate cancer as the most commonly diagnosed neoplasm in Jamaican males. This report examines the association between dietary patterns and risk of prostate cancer in Jamaican men.

**Materials and methods:**

Case-control study of 204 histologically confirmed newly diagnosed prostate cancer cases and 204 individually matched urology clinic controls in Jamaica, 2004 – 2007. Diet was assessed by food frequency questionnaire.

**Results:**

Factor analysis yielded four dietary patterns: (i) a "healthy" pattern of vegetables, fruits and peas and beans, (ii) a "carbohydrate" pattern with high loadings for white bread and refined cereals, (iii) "sugary foods and sweet baked products" pattern and (iv) a "organ meat and fast food pattern" with high loadings for high fat dessert, organ meat, fast food and salty snacks.

Logistic regressions with the individual dietary patterns controlling for potential confounders showed no association between any of the food patterns and risk of prostate cancer. The healthy pattern showed an inverse non-significant association, whereas the carbohydrate pattern was positively and insignificantly related to prostate cancer. Analysis of all food patterns adjusting for each other revealed no association between food patterns and the risk of prostate cancer.

**Conclusion:**

Dietary patterns identified in our sample were not associated with risk of prostate cancer. Further investigations that better define cancer-free subjects and dietary measurements are needed to examine diet and prostate cancer outcomes.

## Introduction

Prostate cancer is the leading cancer site among Jamaican males (30.3%) and the leading cause of cancer mortality (16.5% of total cancer deaths) [1]. A review of age-specific incidence of cancer 1993 – 1997, revealed that age-standardized rate of prostate cancer increased from 36.0/100,000 to 56.4/100,000 [2].

Diet may play a role in the development of prostate cancer. However, studies have been inconsistent in the dietary constituents that affect risk [3]. Another approach has been to examine association between patterns of food consumption and disease risk. In this study factor analysis was used to investigate the association of dietary patterns and prostate cancer in Jamaican men in a matched case-control study.

## Methods

### Sample

The study sample included consecutive men attending urology clinics at 2 major hospitals and private practitioners in Kingston Metropolitan area in Jamaica, 2005 – 2007. This investigation includes 204 histologically confirmed newly diagnosed prostate cancer cases and 204 age-matched urology clinic controls. Thirty-eight cases were excluded from analyses due to the unavailability of a matched control. The study was approved by the Ethics committee of the University of the West Indies and subjects gave written informed consent to participate in the study.

### Data collection

#### Dietary assessment

Dietary intakes were assessed using a food frequency questionnaire developed to assess habitual diets of Jamaican adults. The FFQ was validated against twelve 24-hour recalls over 1 year and showed good agreement. For each food item, subjects provided information on portion size. The FFQ was interviewer-administered.

#### Anthropometry

Body weight (without shoes) was measured in clothing to the nearest 0.1 kg. Height was measured without shoes on a floor standing stadiometer fitted with spirit level, to the nearest 0.1 centimetres. Body mass index was calculated and the World Health Organization's classification was used to determine overweight (BMI >25.00 kg/m^2^) and obesity (BMI ≥ 30.00 kg/m^2^).

#### Other information

Information on demographic and socioeconomic factors, medical history, first-degree family history of prostate cancer and lifestyle were obtained by questionnaire.

### Identification of food patterns

Patterns of food intake were identified by principal component with varimax (orthogonal) rotation to identify non-correlated factors [4]. Individual foods from the FFQ were aggregated into 33 groups, largely based on the macronutrient content of the food and culinary use. Daily intake of the food in grams from each food group was used. The numbers of factors to be retained for the description of food patterns were determined by components with eigenvalue >1 and examination of the scree plots. A four factor solution was identified. Items with factor loadings ≥ 0.4 were used to calculate factor scores for each of the factors.

### Statistical analyses

Factors were divided into tertiles and socio-demographic, anthropometric and dietary characteristics were examined by tertile.

Separate binary logistic regression analyses were performed for each factor to test whether the food pattern was associated with prostate cancer. In all multivariate models, adjustments were made for age, family history of prostate cancer in first degree relatives, education (as a measure of socioeconomic status) smoking, body mass index, alcohol and total energy intake. Analyses were performed using the Statistical Package for Social Sciences (SPSS) version 12. Statistical significance was achieved when p < 0.05.

## Results

### Exclusions

Thirty-six participants only partially completed, or did not complete the FFQ. Using *a priori *exclusion criteria of energy intakes less than 800 or greater than 5500 kilocalories, a further 36 subjects were excluded resulting in a total of 330 subjects for analyses of diet.

### Sample

Men attending urology clinics (n = 972) enrolled in the study were screened by prostate specific antigen (PSA) and/or digital rectal examination (DRE). Seventy percent (681/972) of men screened had an abnormal PSA and/or DRE and 63% (429/681) of these men were biopsied. Prostate cancer was diagnosed in 56.4% (242) of men biopsied. The characteristics of the 204 cases and their matched controls revealed that controls were on average 2 years younger than cases but had similar mean waist circumference and BMI. Reports of a family history of the disease were low in both groups and probably reflect significant underreporting.

### Food patterns

Four food patterns emerged in our sample. Factor 1 showed a "healthy" pattern with high loadings for vegetables, peas and beans and fruits. A "carbohydrate" pattern was seen in factor 2 (white bread and refined cereals, poultry, rice/pasta, starchy roots and tubers) whereas the third factor showed a "sugary foods and sweet baked products" pattern with high loadings for sugary foods, sweet baked goods and non-diet drink. Factor 4 showed an "organ meat and fast food" pattern and consisted of organ meat, fast food and salty snacks. Factor 1 was the dominant food pattern and explained 6.8% of the variance in intake and each of the remaining factors explained between 6.3% and 5.4% of the variance. Together, the 4 factors explained 24.5% of the variance.

There were few socio-demographic and health behaviour characteristics differences by dietary pattern tertiles. Men with high intakes of the carbohydrate pattern showed lower use of vitamins and were less likely to be overweight or obese than men in the lowest tertile. Men in the highest tertile of the sugary foods and sweet baked goods pattern were more likely to be current smokers and were less likely to have tertiary education. Men with high intakes of the organ meat and fast food pattern were more likely to be obese.

Odds ratios (CI) for the risk of prostate cancer according to tertiles of the food pattern scores are shown in Table 1. No association was found between any of the individual food patterns and risk of prostate cancer. As an individual's diet would comprise all food patterns we analysed the four food patterns adjusting for each other for their association with prostate cancer. In this analysis no association was found between the food patterns and risk of prostate cancer.

**Table 1:** Multivariate OR (CI) for Tertiles of 4 Food Patterns associated with Prostate Cancer in Jamaican men

	**Tertiles of dietary pattern score**		
	**Tertile 1 (low)**	**Tertile 2**	**Tertile 3 (high)**
**Factor 1: Vegtables, peas, beans & fruits (Healthy)**			
Total prostate cancer cases	111	108	110
Multivariate OR (CI)^a^	1.00 (reference)	0.72 (0.39 – 1.32)	0.84 (0.44 – 1.59)
**Factor 2: Carbohydrate**			
Total prostate cancer cases	110	109	111
Multivariate OR (CI)^a^	1.00 (reference)	1.28 (0.70 – 2.34)	1.20 (0.58 – 2.48)
**Factor 3: Sugary foods & sweet baked goods**			
Total prostate cancer cases	102	113	115
Multivariate OR (CI)^a^	1.00 (reference)	0.88 (0.49 – 1.62)	0.75 (0.40 – 1.38)
**Factor 4: Organ meat and fast food**			
Total prostate cancer cases	104	117	109
Multivariate OR (CI)^a^	1.00 (reference)	1.31 (0.73 – 2.33)	0.96 (0.53 – 1.76)
Four dietary patterns:^a^			
**Vegetables, Peas & Beans, fruits**	1.00 (reference)	0.67 (0.36 – 1.27)	0.81 (0.43 – 1.58)
**Carbohydrate**	1.00 (reference)	1.27 (0.69 – 2.36)	1.16 (0.55 – 2.47)
**Sugary foods & sweet baked goods**	1.00 (reference)	0.88 (0.48 – 1.63)	0.72 (0.38 – 1.35)
**Organ meat and fast food**	1.00 (reference)	1.26 (0.70 – 2.27)	0.88 (0.47 – 1.63)

^a ^All models adjusted for age, family history of prostate cancer, education, body mass index, smoking, alcohol and total energy intake.

## Discussion

The principal component analysis of our matched case-control study identified 4 food patterns: a "vegetables, peas, beans and fruits (healthy), a "carbohydrate" pattern, a "sugary foods and baked goods" pattern and an "organ meat and fast food" pattern. There were no significant associations between food patterns and risk of prostate cancer. Further examination of a model including all four food patterns also showed no evidence of an association with prostate cancer.

The absence of an association of dietary patterns with prostate cancer in our study is consistent with a recent prospective study of the U.S. health professionals that showed that neither the prudent or western dietary patterns were associated with the risk of prostate cancer [5]. In contrast, Tseng et al [6], suggested that higher intakes of a Southern pattern (cornbread, grits, sweet potatoes, okra, beans and rice) was protective, whereas Walker et al [7] reported that refined grain products, processed meats or red and organ meats increased the risk of prostate cancer. Higher intakes of healthy pattern in our study though suggestive, showed no evidence for an inverse association with risk of prostate cancer.

The absence of a relationship between dietary patterns and prostate cancer may be due to insufficient variability of dietary intakes or inaccurate measurement of the underlying pattern. Furthermore, associations may be obscured if undiagnosed prostate cancer cases were included as cancer-free men for comparisons.

## Conclusion

Our study suggested that the dietary patterns identified in our sample were not associated with risk of prostate cancer. Further investigations that better define cancer-free subjects and diet are needed to examine the contribution of diet to prostate cancer outcomes.

## Competing interests

The authors declare that they have no competing interests.

## Authors' contributions

All authors contributed to the design of the study. MJ, CS, NM and FB were responsible of the execution of the study. MJ conducted the analysis and drafted the manuscript. SW assisted with the analyses and contributed to the interpretation of results. All authors critically reviewed the manuscript.
